# Differential survival and tolerance mechanisms of *Bacillus subtilis* and *Salmonella Enteritidis* under slightly acidic electrolyzed water stress

**DOI:** 10.1128/aem.01817-25

**Published:** 2026-01-12

**Authors:** Jiahong Han, Guosheng Zhang, Yao Zang, Congfang Hong, Yingjie Feng, Shan Bing, Nengshui Ding, Guoyun Wu, Hongxiang Wu, Haojie Zhu, Yitian Zang

**Affiliations:** 1 Key Laboratory of Animal Health and Safe Production of Nanchang, Jiangxi Agricultural University 91595, Jiangxi, China; 2 Jiangxi Agricultural Technology Extension Center, Jiangxi, China; Anses, Maisons-Alfort Laboratory for Food Safety, Maisons-Alfort, France

**Keywords:** slightly acidic electrolyzed water, *Bacillus subtilis*, *Salmonella enteritidis*, selective bactericidal activity, tolerance mechanisms

## Abstract

*Salmonella Enteritidis* (*S. Enteritidis*) is a major foodborne pathogen, and its effective control is critical for food safety. Although slightly acidic electrolyzed water (SAEW) is widely adopted as an eco-friendly bactericidal agent in food systems, its impact on beneficial microbiota remains poorly characterized, despite their importance for food quality. To address this gap, we systematically compared SAEW tolerance between *Bacillus subtilis* (*B. subtilis,* a model probiotic) and *S. Enteritidis* in monoculture and co-culture systems. Notably, *B. subtilis* retained viability at 70 mg/L ACC (from 7.82 to 3.99 log CFU/mL, *P* < 0.05), whereas *S. Enteritidis* was completely inactivated at 40 mg/L. In co-culture, *B. subtilis* maintained consistently higher viable counts (*P* < 0.05), demonstrating a significant survival advantage under SAEW stress. Furthermore, *B. subtilis* demonstrated rapid recovery in co-culture, attaining 8.0 log CFU/mL within 32 h post-exposure, while *S. Enteritidis* was eradicated. Mechanistically, SAEW disrupted membrane integrity in both strains but triggered divergent stress responses: *S. Enteritidis* exhibited significant ROS accumulation (*P* < 0.05), ATP depletion (*P* < 0.05), and suppressed the activities of key antioxidant enzymes (*P* < 0.05). Conversely, *B. subtilis* showed significant upregulation of these enzymes (SOD, CAT, GSH-Px, *P* < 0.05) with stable ROS levels. Consequently, SAEW enables selective pathogen inactivation while preserving probiotic strains, supporting its targeted application in food systems.

**IMPORTANCE:**

The increasing adoption of slightly acidic electrolyzed water (SAEW) as an eco-friendly disinfectant in food safety highlights the need for a deeper understanding of its selective bactericidal mechanisms. This study addresses a critical gap in the literature by demonstrating that SAEW effectively targets harmful pathogens, such as *Salmonella Enteritidis*, while preserving beneficial probiotics, such as *Bacillus subtilis*. By elucidating the differential stress responses of these microorganisms, our findings provide valuable insights into the ecological dynamics of food systems. The ability of SAEW to selectively inactivate pathogens without disrupting beneficial microbiota supports its targeted application in enhancing food safety and quality. This research not only advances the scientific understanding of SAEW’s mechanisms but also has practical implications for developing safer food preservation methods, ultimately contributing to public health and food security.

## INTRODUCTION

*Salmonella* is among the most significant foodborne pathogens, contributing substantially to global illness and mortality. Annually, it is responsible for an estimated 93.8 million illnesses and 155,000 deaths worldwide (1, 2). Despite ongoing control efforts, *Salmonella enterica* serovar Enteritidis (*S. Enteritidis*), in particular, remains a major concern, as evidenced by outbreaks causing fatalities across 18 European countries between 2016 and 2020, especially among vulnerable populations (3). Thus, effective disinfection strategies are essential for preventing *S. Enteritidis* contamination in food processing environments. Traditional disinfection approaches, such as thermal processing or chemical sanitizers, have well-known limitations (4, 5). Heat treatments may compromise food quality, while conventional chlorine-based disinfectants may leave harmful residues, raising food safety concerns. Accordingly, there is a growing interest in developing alternative disinfectants that are both effective and residue-free (4, 6).

Slightly acidic electrolyzed water (SAEW), with a typical pH range of 5.0–6.5, has emerged as a promising disinfection strategy (7, 8). Produced through the electrolysis of dilute hydrochloric acid or sodium chloride, SAEW is characterized by low corrosiveness, broad-spectrum antimicrobial activity, and environmental compatibility. Its primary bactericidal component, hypochlorous acid (HClO), efficiently inactivates a wide range of microorganisms, including *Salmonella* spp. (9). Our prior research has also confirmed that available chlorine concentrations (ACCs), rather than treatment time or ORP, is the primary determinant of SAEW’s bactericidal efficacy (10). SAEW has thus been widely explored for use in food disinfection, surface sanitation, and environmental hygiene (11, 12).

However, SAEW’s broad-spectrum nature raises concerns regarding non-selective microbial inactivation. In food production environments, complex microbial ecosystems often include not only pathogens but also beneficial microorganisms such as *B. subtilis* (13, 14). Disrupting these ecological communities may reduce microbial diversity and stability, potentially impairing long-term pathogen suppression and food fermentation quality (15, 16). This challenge demands selective microbial control strategies, particularly disinfection technologies capable of targeted pathogen elimination without disrupting beneficial microbes. Such concepts have been discussed in the context of microbial ecological resilience and probiotic conservation (14, 16).

*B. subtilis* is a spore-forming, probiotic bacterium known for its strong adaptability, safety, and antagonism against pathogens. It is widely used in agriculture, fermentation, and food preservation ( 17, 18). Notably, prior studies have reported its strong tolerance to chlorine-based disinfectants (19). For instance, Hao et al. (19) showed that *B. subtilis* had higher survival rates than *Escherichia coli* and *Staphylococcus aureus* (*S. aureus*) under SAEW exposure. Zhang et al. (20) further observed that even under UV-sodium hypochlorite combined treatment, *B. subtilis* maintained viability at low ACC. Such findings suggest that *B. subtilis* possesses physiological features—such as robust membrane structures, oxidative stress responses, and repair mechanisms—that confer resistance to oxidative damage induced by chlorine-based agents (21–23). Despite these insights, few studies have examined whether SAEW exhibits selective inactivation of pathogens, such as *S. Enteritidis*, while sparing beneficial species, such as *B. subtilis*, in co-culture settings. Furthermore, the underlying physiological mechanisms of such differential tolerance, particularly regarding membrane integrity, energy metabolism (e.g., ATP), and oxidative stress responses, remain poorly understood. Exploring these mechanisms will provide valuable guidance for applying SAEW in ways that minimize disruption to beneficial microbial communities.

Based on these considerations, we hypothesized that *S. Enteritidis* and *B. subtilis* display markedly different responses to SAEW stress, with *S. Enteritidis* being more susceptible. This differential tolerance may be reflected in their survival rates, interspecies dynamics, and physiological stress responses. To test this hypothesis, the present study addressed four specific objectives: (i) evaluate the bactericidal effects of varying ACC levels on each strain in single-culture systems and model their survival trends; (ii) assess interspecies differences in tolerance under co-culture conditions exposed to SAEW; (iii) monitor the dynamic changes in bacterial viability after SAEW treatment to determine strain-specific recovery patterns; and (iv) elucidate the underlying tolerance mechanisms by analyzing membrane integrity, ATP depletion, ROS accumulation, and antioxidant enzyme activity. This study aims to explore the selective microbial control potential of SAEW, particularly focusing on its differential effects on *S. Enteritidis* and beneficial microbes like *B. subtilis*. Understanding these mechanisms will help optimize SAEW application in food safety practices, preserving both pathogen control and microbial diversity essential for food fermentation.

## MATERIALS AND METHODS

### Preparation of bacterial cultures

*S. Enteritidis* (BNCC-103134) and *B. subtilis* (BNCC-109047) lyophilized strains were purchased from Bena Culture Collection Center (Henan, China). Both strains were activated in 25 mL tryptic soy broth (TSB, Beijing Aoboxing Bio-Tech Co., Ltd., Beijing, China) at 37°C with shaking (150 rpm) for 24 h. Activated cultures were mixed 1:1 with 40% glycerol and stored at −80°C until use. Before the experiments, frozen stock vials were rapidly thawed at 37°C for 2 min, inoculated into 25 mL TSB, and cultured at 37°C for 24 h with shaking. The cultures were centrifuged (4,000 rpm, 4°C, 10 min), the pellets were resuspended in 25 mL sterile 0.1% peptone water (Beijing Aoboxing Bio-Tech Co., Ltd., Beijing, China) and adjusted to approximately 8–9 log CFU/mL. Cell concentrations were confirmed by plating serial dilutions onto nutrient agar (NA, Qingdao Hope Bio-Technology Co., Ltd., Shandong, China), followed by incubation at 37°C for 24 h.

### Preparation of SAEW

SAEW was generated using an electrolyzed water device (V9, Zhongshan Lvfuren Electrical Appliance Co., Ltd., Guangdong, China). Briefly, 2 L of 1 g/L NaCl solution supplemented with 0.35 mL concentrated HCl was electrolyzed for 1.5 h, then immediately diluted with sterile deionized water to obtain solutions with different ACCs (10, 20, 30, 40, 50, and 70 mg/L). The pH (6.16–6.21) and oxidation-reduction potential (ORP, 884–896 mV) were measured using a pH meter (HM-30R, Shanghai Leichi Co., Ltd., Shanghai, China) and an ORP meter (SX-630, Shanghai Sanxin Co., Ltd., Shanghai, China), respectively. The ACC values were determined with a digital chlorine analyzer (YXL-1A, Shanghai Haizheng Electronic Technology Co., Ltd., Shanghai, China).

### Determination of single-strain survival under SAEW stress at different ACC concentrations

**Fig 1:**
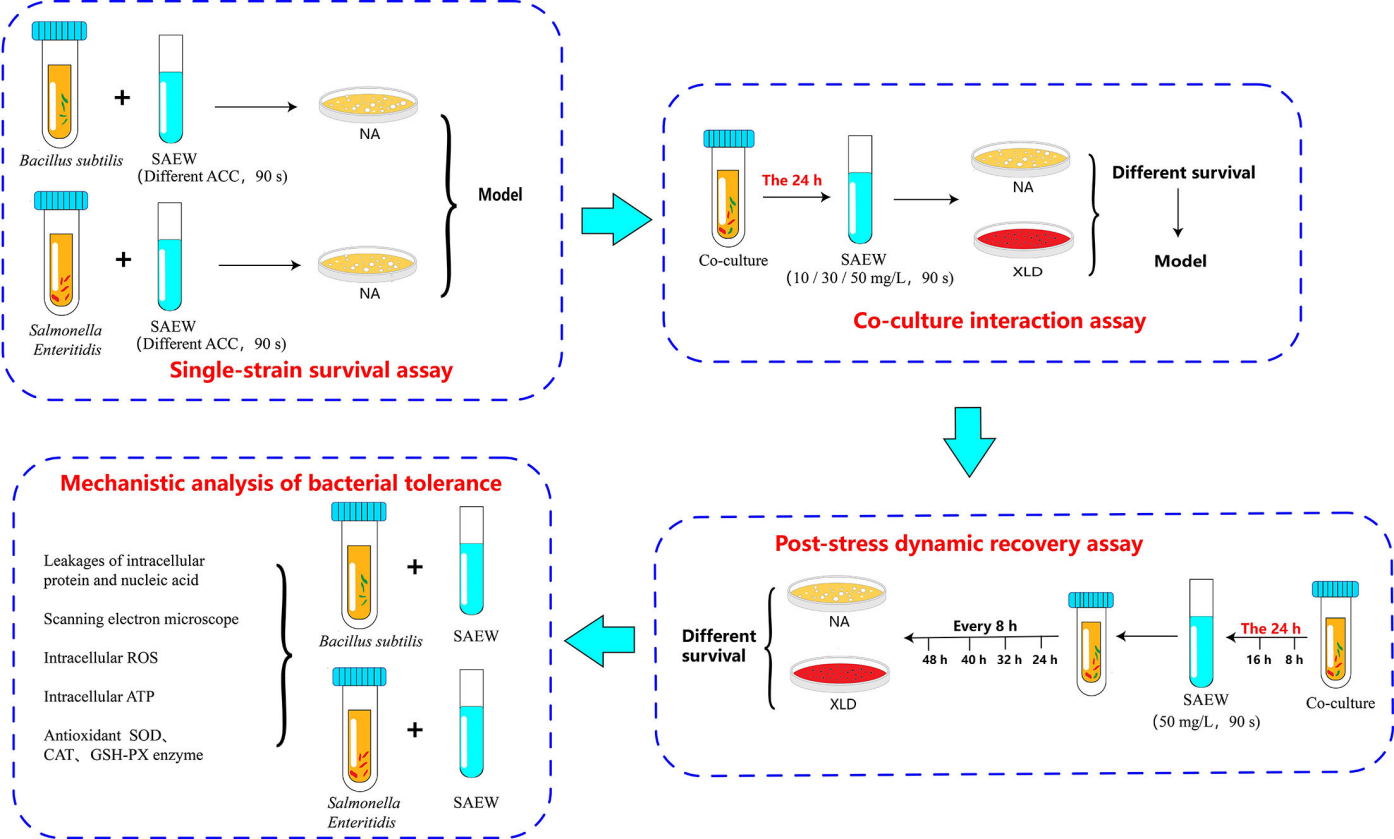
Experimental workflow illustrating differential survival and tolerance mechanisms of *Bacillus subtilis* and *Salmonella Enteritidis* under slightly acidic electrolyzed water (SAEW) stress. (Top left) Single-strain survival assay, in which each bacterium was treated with various concentrations of SAEW (available chlorine concentration, ACC) for 90 s to establish baseline tolerance profiles. (Top right) Co-culture interaction assay, in which the two strains were first co-cultured for 24 h, followed by SAEW treatment (10, 30, or 50 mg/L, 90 s) to assess immediate survival differences using selective media (NA for *B. subtilis*, XLD for *S. Enteritidis*). (Bottom right) Post-stress dynamic recovery assay, in which the 24-hour co-cultured bacteria were treated with SAEW (50 mg/L, 90 s) and then cultured continuously for 48 h, with viable counts measured every 8 h to evaluate recovery capacity. (Bottom left) Mechanistic analysis of bacterial tolerance involving scanning electron microscopy, intracellular ATP and ROS quantification, protein and nucleic acid leakage, and measurements of antioxidant enzymes (SOD, CAT, and GSH-Px).

The overall experimental workflow is illustrated in Fig. 1. A single-factor experiment was initially conducted to select suitable ACCs (10, 20, 30, 40, 50, and 70 mg/L) of SAEW. Briefly, 1 mL bacterial suspension (*S. Enteritidis* or *B. subtilis*, ~9.00 ± 0.02 log CFU/mL) was added separately into 9 mL SAEW solutions with different ACCs and treated for 90 s. The reaction was stopped immediately by adding 1 mL sodium thiosulfate. Sterile saline was used instead of SAEW as a control. After treatment, bacterial suspensions were serially diluted (10-fold) in sterile 0.1% peptone water. Aliquots (0.1 mL) of appropriate dilutions were plated in triplicate onto NA plates and incubated at 37°C for 24 h for colony enumeration. All NA plates from control treatments (without bacterial inoculation) exhibited no bacterial growth, confirming the effectiveness of aseptic techniques.

### Measurement of bacterial counts in co-culture under SAEW stress at different ACC concentrations

As the subsequent step in the workflow illustrated in Fig. 1, this section evaluates bacterial counts in co-culture systems. This experiment aimed to evaluate the dynamic changes in bacterial counts of *S. Enteritidis* and *B. subtilis* in co-culture systems exposed to SAEW with different available chlorine concentrations (ACCs). Initially, the bacterial suspensions of *S. Enteritidis* and *B. subtilis* were each adjusted to approximately 3.00 ± 0.02 log CFU/mL. Equal volumes (0.5 mL) of the two bacterial suspensions were combined into 9 mL TSB medium and incubated at 37°C for 24 h.

After incubation, 1 mL aliquots of co-cultured suspension were treated separately with 9 mL SAEW at different ACC concentrations (10, 30, and 50 mg/L) for 90 s. Immediately after treatment, the reaction was neutralized by adding 1 mL sodium thiosulfate. Co-culture suspensions treated with sterile saline instead of SAEW were used as controls.

Following treatment, the suspensions were serially diluted (10-fold) in sterile 0.1% peptone water. Aliquots (0.1 mL) from each dilution were spread-plated in triplicate on both NA and Xylose Lysine Deoxycholate (XLD) agar (Qingdao Hope Bio-Technology Co., Ltd., Shandong, China) plates and incubated at 37°C for 24 h. The total bacterial colonies (*S. Enteritidis + B. subtilis*) were enumerated on NA plates, while colonies of *S. Enteritidis* were selectively counted on XLD agar based on their distinct colony morphology. The counts of *B. subtilis* were calculated by subtracting *S. Enteritidis* colony counts (on XLD agar) from the total counts (on NA agar).

### Measurement of bacterial population dynamics over time after SAEW stress

Following the co-culture evaluation (as shown in Fig. 1), this section assesses population dynamics during recovery. This experiment aimed to evaluate the dynamic changes in bacterial populations of *S. Enteritidis* and *B. subtilis* during co-culture after SAEW stress and the subsequent recovery phase. The experiment included three sequential phases:

(i) Co-culture growth phase: Equal volumes (0.5 mL each) of *S. Enteritidis* and *B. subtilis* suspensions (initially adjusted to four log CFU/mL) were mixed in 9 mL TSB and co-incubated at 37°C for 24 h. Samples were collected every 8 h, serially diluted, plated onto NA and XLD agar, and incubated at 37°C for 24 h to determine bacterial counts at each interval.

(ii) SAEW treatment phase: After 24 h co-culture, 1 mL of bacterial suspension was transferred into 4 mL SAEW solution (50 mg/L ACC) for 90 s. The reaction was immediately terminated by adding 1 mL sodium thiosulfate. Surviving bacterial numbers were enumerated as described above.

(iii) Post-treatment recovery phase: Following SAEW treatment, 1 mL of bacterial suspension was re-inoculated into fresh 9 mL TSB and cultured again at 37°C for another 24 h. Samples were collected every 8 h for bacterial enumeration, enabling assessment of bacterial recovery dynamics after SAEW stress. Each experimental group was conducted independently in triplicate.

### Analysis of bacterial tolerance mechanisms under SAEW stress

As the final analytical step in the workflow illustrated in Fig. 1, this section examines tolerance mechanisms. SAEW with an ACC of 30 mg/L was applied for 90 s to evaluate the tolerance mechanisms of *S. Enteritidis* and *B. subtilis*. Untreated bacterial suspensions served as the control group. After treatment, bacterial samples were immediately collected and subjected to the following analyses:

#### Intracellular protein leakage measurement

After SAEW treatment, bacterial suspensions were centrifuged at 4,000 rpm for 10 min at 4°C, and the supernatant was collected. Intracellular protein leakage was quantified using a commercial BCA Protein Assay Kit (GenStar Biosolutions Co., Ltd., Jiangsu, China) according to the manufacturer’s protocol. Briefly, 20 μL of supernatant samples were mixed with 200 μL of BCA working reagent in microplate wells. After incubation at 37°C for 30 min, absorbance at 562 nm was measured using a microplate reader (Spectra MaxM5 190, Molecular Devices Shanghai Corporation Co., Ltd., Shanghai, China). Protein concentration was calculated based on a standard BSA curve.

#### Intracellular nucleic acid leakage measurement

After SAEW treatment, bacterial samples were centrifuged at 12,000 rpm for 3 min, and 2 μL of supernatant was analyzed using a UV spectrophotometer (752 N, Hangzhou Jingke Instrument Co., Ltd., Zhejiang, China) to determine nucleic acid leakage by measuring absorbance at 260 nm.

#### Scanning electron microscopy (SEM) analysis

After SAEW treatment, bacterial suspensions were centrifuged at 6,000 rpm (4°C, 5 min), washed twice with PBS buffer, and fixed overnight at 4°C with 1 mL of 2.5% glutaraldehyde. Samples were then washed three times with PBS buffer, followed by a graded ethanol dehydration series (30%, 50%, 70%, 80%, 90%, and 100% ethanol, each for 15–20 min). After dehydration, bacterial cells were resuspended in 0.5 mL absolute ethanol. A 10 μL aliquot was transferred onto a silicon wafer and air-dried naturally. The dried samples were then sputter-coated with platinum and observed under a scanning electron microscope (Regulus 8100, Japan Electronics Company, Tokyo, Japan) at an accelerating voltage of 5.0 kV and magnification of 20,000× to investigate cellular morphological changes.

#### Intracellular ATP assay

Following SAEW treatment, bacterial suspensions were centrifuged at 4,000 rpm (4°C) for 10 min. Pellets were washed with physiological saline and disrupted by ultrasonication in an ice bath for 10 min. The lysate was centrifuged at 8,000 rpm (4°C) for 15 min, and 10 μL of the supernatant was analyzed for intracellular ATP content using an ATP assay kit (Suzhou Comin Biotechnology Co., Ltd., Jiangsu, China), following the manufacturer’s instructions. Briefly, 50 μL of prepared samples were incubated at 37°C for 30 min, after which 200 μL of ATP color-developing reagent was added and incubated for an additional 20 min at 37°C. Absorbance was measured at 700 nm using a microplate reader, and ATP concentrations were calculated from a standard curve.

#### Reactive oxygen species (ROS) assay

SAEW-treated bacterial samples were centrifuged at 2000 rpm (4°C) for 5 min, washed once with PBS buffer, and resuspended in 10 μM fluorescent probe DCFH-DA solution (ROS Assay Kit, NanJing JianCheng Bioengineering Institute, Jiangsu, China). Samples were incubated at 37°C in darkness for 40 min. Fluorescence intensity indicating ROS accumulation was measured using a fluorescence microplate reader (357-701430T, Variskon Flash, Thermo Fisher Scientific, Shanghai, China) at excitation/emission wavelengths of 502 nm and 530 nm, respectively.

#### Antioxidant enzyme activity assay

Following SAEW treatment, bacterial suspensions were centrifuged at 4,000 rpm (4°C) for 10 min. Pellets were resuspended in physiological saline and disrupted by ultrasonication (ice bath, 10 min). The lysate was then centrifuged at 8,000 rpm (4°C) for 15 min to obtain the supernatant. Superoxide dismutase (SOD), catalase (CAT), and glutathione peroxidase (GSH-Px) activities in the supernatant were measured using corresponding commercial assay kits (NanJing JianCheng Bioengineering Institute, Jiangsu, China), following the manufacturers’ protocols. For each enzyme, 20 μL of the sample was mixed with the provided working reagents, incubated at 37°C, and absorbance was measured using a microplate reader (Spectra MaxM5 190, Molecular Devices Shanghai Corporation Co., Ltd., Shanghai, China) at 560 nm (SOD), 405 nm (CAT), and 412 nm (GSH-Px), respectively. Enzyme activities were calculated based on standard curves and expressed as U/mg protein.

### Statistical analysis

All experiments were independently performed in triplicate, and data were expressed as mean ± standard deviation (SD). Statistical analyses were performed using SPSS 26.0 (IBM, USA) and Origin 9.0 (OriginLab, USA). Linear regression analysis was used to evaluate trends in bacterial counts under single-strain and co-culture conditions at different ACCs of SAEW. Logistic regression analysis was conducted to describe bacterial population dynamics over time following SAEW treatment. One-way analysis of variance (ANOVA), followed by Tukey’s multiple comparison test, was applied to identify significant differences among groups, with statistical significance defined as *P* < 0.05.

## RESULTS AND DISCUSSION

### Survival of single strains under SAEW stress at different ACC concentrations

**Fig 2:**
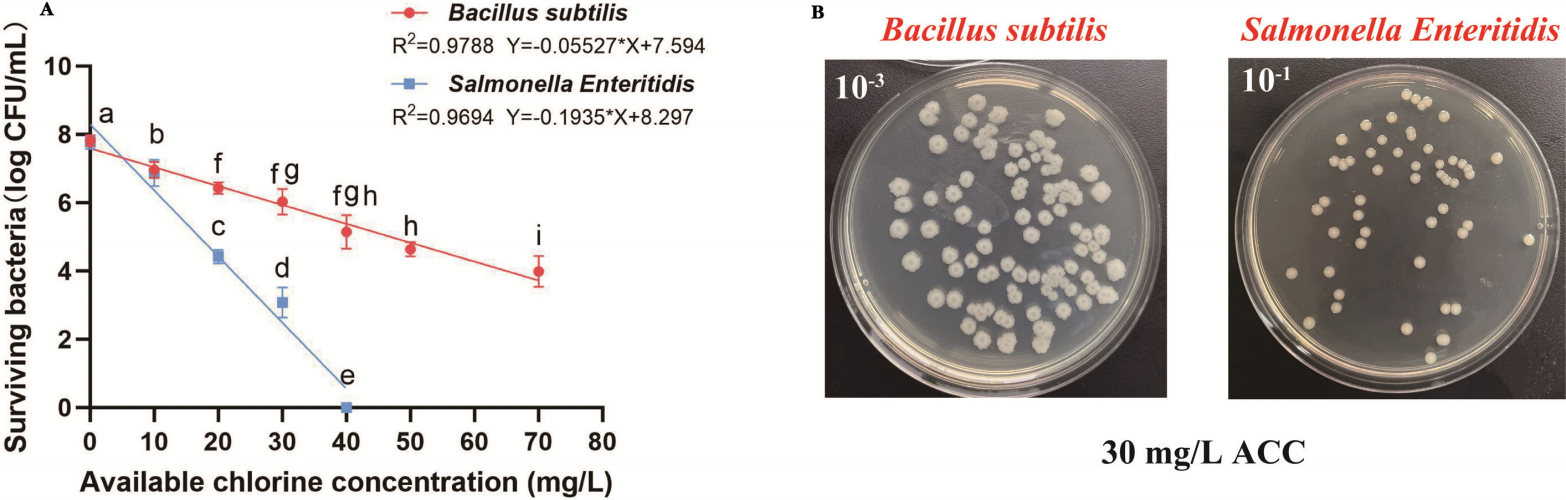
Survival of *Bacillus subtilis* and *Salmonella Enteritidis* under slightly acidic electrolyzed water (SAEW) stress in single-strain assays. (**A**) The two strains were treated with SAEW at various available chlorine concentrations (ACCs) for 90 s. The linear regression models for bacterial survival are presented. Data are expressed as mean ± SD (*n* = 3). Different lowercase letters indicate statistically significant differences between groups (*P* < 0.05). (**B**) Representative colony morphology of each strain grown on nutrient agar (NA) after treatment with 30 mg/L ACC.

To assess inherent differences in resistance to SAEW, *B. subtilis* and *S. Enteritidis* were separately cultured and exposed to a range of ACCs. As shown in Fig. 2, both strains exhibit significant viability loss with increasing ACC (*P* < 0.05), consistent with previous studies (9, 24). Our prior research has confirmed that ACC, rather than treatment time or ORP, is the primary determinant of SAEW’s bactericidal efficacy (10). This is largely due to HClO, the dominant active species at near-neutral pH, which exhibits higher oxidative potential and lower volatility compared to hypochlorite ions and chlorine gas (10).

However, the magnitude and pattern of viability reduction differed markedly between the two strains. The viable count of *S. Enteritidis* dropped sharply from 7.78 to 3.08 log CFU/mL at 30 mg/L, and complete inactivation was observed at 40 mg/L. In contrast, *B. subtilis* declined more gradually, from 7.82 to 3.99 log CFU/mL even at 70 mg/L. At all ACC levels above 10 mg/L, *B. subtilis* maintained significantly higher viable counts, suggesting superior tolerance to SAEW-induced oxidative stress.

Linear regression analysis further supported this distinction. The models for *B. subtilis* and *S. Enteritidis* yielded high coefficients of determination (*R*² = 0.9788 and 0.9694, respectively), indicating strong model fits. Notably, the absolute value of the slope for *S. Enteritidis* (−0.1935) was approximately 3.5 times greater than that for *B. subtilis* (−0.0553), reflecting a substantially faster rate of inactivation per unit ACC increase. Similar findings have been reported by Hao et al. (19), who showed that SAEW at 25 mg/L completely eliminated *E. coli* and *S. aureus*, but had limited efficacy against *B. subtilis.* Wang et al. (25) likewise observed lower inactivation constants (K values) for *B. subtilis* (0.0488) than for *E. coli* (0.2897) and *S. aureus* (0.1106) under combined UV and chlorine treatment.

These data indicate that the differential tolerance may be rooted in fundamental physiological distinctions. As a Gram-positive bacterium, *B. subtilis* possesses a thick peptidoglycan layer and a less permeable cell envelope, which likely confer structural resistance to membrane-targeting agents (21, 23). In addition, it exhibits strong oxidative stress defense mechanisms, including elevated levels of SOD and CAT, and may initiate sporulation-related protective responses under extreme conditions (22). In contrast, the thinner outer membrane and higher permeability of Gram-negative *S. Enteritidis*, coupled with its comparatively weaker antioxidant systems, likely render it more vulnerable to oxidative disruption, membrane leakage, and cellular damage during SAEW exposure (9, 24). Still, the precise mechanisms underlying this differential resistance remain to be clarified.

While these results establish a clear difference in single-strain SAEW tolerance, microbial behavior in real-world environments is often shaped by interspecies interactions (26). Microorganisms rarely exist in isolation; rather, they share ecological niches and can modulate each other’s stress responses through competition, metabolic interference, or signaling (27). Therefore, whether the resistance advantage of *B. subtilis* remains observable in the presence of *S. Enteritidis* requires further investigation. To address this, we next established a co-culture model and exposed it to the same ACC gradients, aiming to determine whether the strain-specific tolerance identified here persists in a mixed microbial environment.

### Survival of co-cultured bacteria under SAEW stress at different ACC concentrations

**Fig 3:**
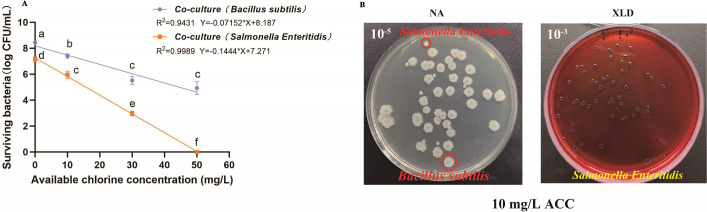
Survival of *Bacillus subtilis* and *Salmonella Enteritidis* following slightly acidic electrolyzed water (SAEW) treatment under co-culture conditions. (**A**) Mixed cultures incubated for 24 h were treated with SAEW at 10, 30, and 50 mg/L available chlorine concentration for 90 seconds. The surviving bacteria were enumerated using selective media and expressed as log CFU/mL. Different lowercase letters indicate significant differences between groups (*P* < 0.05). (**B**) Representative colony morphology of co-cultured bacteria treated with 10 mg/L ACC. Colonies grown on nutrient agar (NA, left) show morphological differences between *B. subtilis* and *S. Enteritidis*, while XLD agar (right) selectively visualizes *S. Enteritidis* colonies.

To evaluate whether the differential tolerance to SAEW observed under single-strain conditions is preserved in a mixed microbial environment, *B. subtilis* and *S. Enteritidis* were co-cultured for 24 h and then treated with SAEW at varying ACCs. As shown in Fig. 3, the viability of both strains decreases significantly with increasing ACC (*P* < 0.05). However, *B. subtilis* consistently maintained significantly higher viable counts than *S. Enteritidis* at all tested concentrations. At 10 mg/L ACC, *B. subtilis* and *S. Enteritidis* reached 7.40 and 5.96 log CFU/mL, respectively. This difference widened at 30 mg/L (5.52 vs 2.97 log CFU/mL), and at 50 mg/L (4.94 vs 0 log CFU/mL), *S. Enteritidis* was completely inactivated, while *B. subtilis* retained 4.94 log CFU/mL.

Linear regression analysis confirmed the persistence of this difference. The absolute value of the slope for *S. Enteritidis* (−0.1444) was approximately double that of *B. subtilis* (−0.0715), indicating a faster rate of viability loss per unit increase in ACC. These results demonstrate that the strain-specific resistance pattern previously observed under isolated conditions is not masked in co-culture systems.

Previous research has established that the broad-spectrum antimicrobial activity of SAEW remains stable in the presence of coexisting microorganisms (10, 11). This finding aligns with the results of the present study: in a planktonic co-culture system, the presence of *B. subtilis* did not significantly attenuate the inactivation efficacy of SAEW against *S. Enteritidis*. Furthermore, interspecific interactions (e.g., competition or antagonism) did not obscure the inherent tolerance disparity between the two bacterial species.

Conversely, specific environments may confer relative bactericidal selectivity to SAEW. For example, Zhao et al. (28) observed that during vaginal cleansing applications, acidic electrolyzed water effectively eliminated pathogenic anaerobes while relatively preserving acid-tolerant lactobacilli. This suggests that specific physiological structures (e.g., a thick peptidoglycan layer) or stress resistance mechanisms may partially counteract electrolyzed water-induced damage (28, 29). In this study, *B. subtilis* exhibited significantly higher survival rates than *S. Enteritidis* at 50 mg/L ACC. However, *B. subtilis* itself still incurred losses exceeding 3 log units. This indicates that its tolerance advantage is dose dependent, the specific mechanisms of which warrant further investigation. Additionally, although *B. subtilis* is capable of producing antimicrobial lipopeptides (e.g., surfactin, fengycin) (30), the strong correlation observed in this study between SAEW dose and survival rate demonstrates that direct oxidative damage by SAEW constitutes the primary lethal mechanism. Under these conditions, the contribution of interspecies antagonism may be masked.

Together, these results reinforce the notion that SAEW-induced inactivation is primarily driven by species-specific oxidative tolerance rather than by ecological competition. However, the notable reduction in *B. subtilis* populations also raises concerns regarding its recovery potential and post-treatment functionality. To further address this, we next investigated the dynamic changes in viability of both strains during post-treatment recovery, aiming to assess the resilience and competitive regrowth patterns under co-culture conditions.

### Dynamic responses of co-cultured bacteria to SAEW treatment

To evaluate microbial responses to disinfection at the stationary phase, *B. subtilis* and *S. Enteritidis* were co-cultured for 24 h to reach a stable high-density state, followed by treatment with SAEW (50 mg/L ACC, 90 s). Post-treatment viability was monitored over a 24-hour period to assess species-specific recovery capacities.

**Fig 4:**
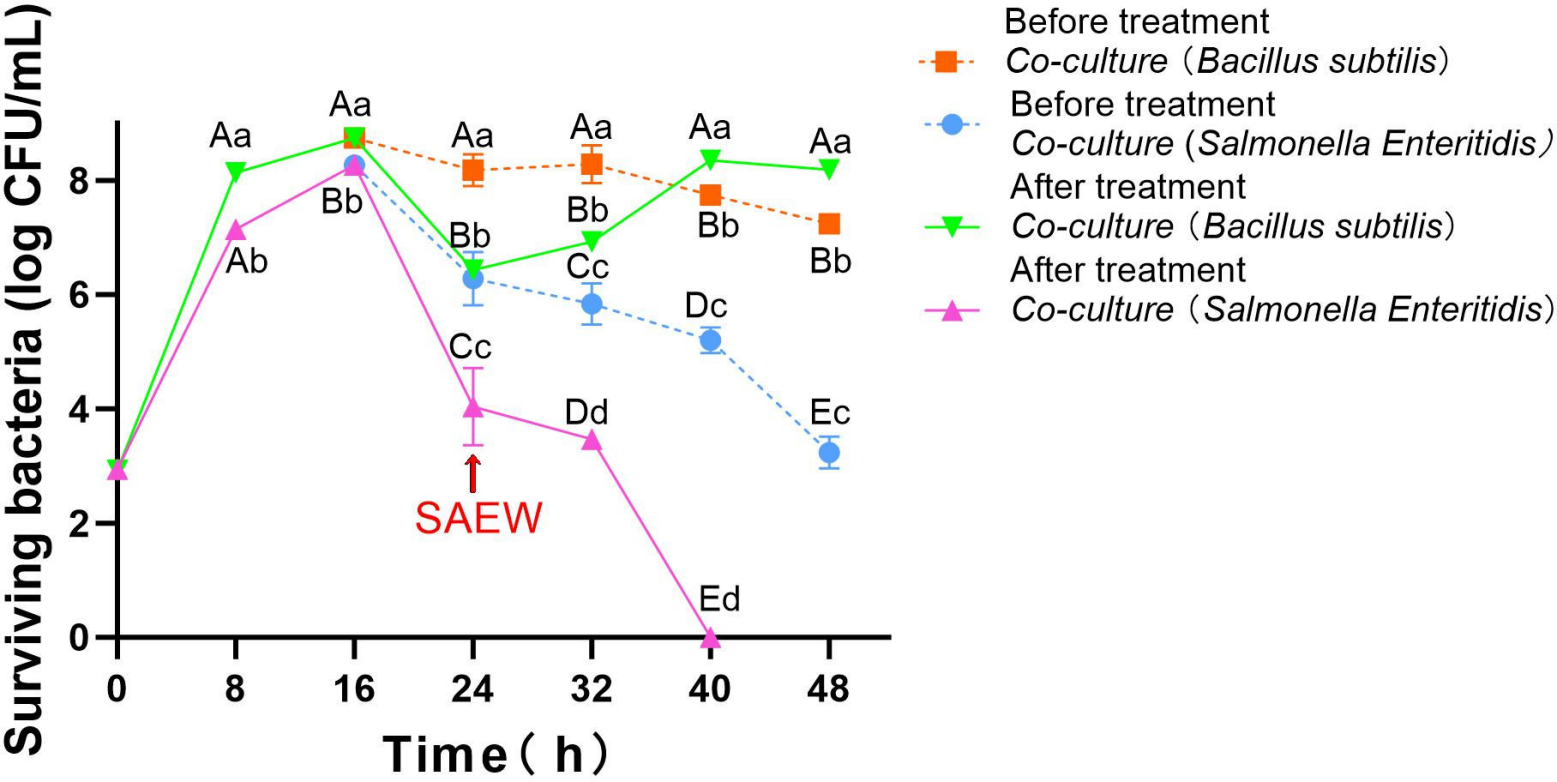
Dynamic changes in viability of *Bacillus subtilis* and *Salmonella Enteritidis* under co-culture conditions following slightly acidic electrolyzed water (SAEW) treatment (50 mg/L) at 24 h. After 24 h of co-culture, mixed bacterial suspensions were treated with SAEW (50 mg/L, 90 s), and then incubated for 48 h. Viable counts of each strain were determined every 8 h on selective media and expressed as log CFU/mL. Different capital letters indicate significant differences among the same bacterial strain at different times (*P* < 0.05). Different lowercase letters indicate significant differences among different bacterial strains at the same time (*P* < 0.05).

As shown in Fig. 4, both bacterial populations reach peak levels prior to SAEW exposure, with *B. subtilis* and *S. Enteritidis* reaching 8.74 and 8.27 log CFU/mL, respectively. Note that the initial bacterial concentrations in this co-culture setup were adjusted to ~4 log CFU/mL for each species (compared to ~3 log CFU/mL in “Survival of co-cultured bacteria under SAEW stress at different ACC concentrations,” above) to facilitate observation of recovery patterns, prevent complete inactivation of *S. Enteritidis*, and enable rapid attainment of a stable state for long-term viability assessment. Immediately after SAEW treatment, significant reductions were observed: *S. Enteritidis* declined to 4.04 log CFU/mL, while *B. subtilis* dropped to 6.43 log CFU/mL. The observed variation in SAEW efficacy (~7 log in “Survival of co-cultured bacteria under SAEW stress at different ACC concentrations,” above, vs ~2 log reduction) highlights the impact of initial inoculum density on antimicrobial outcomes in co-cultures. Higher starting concentrations may promote denser biofilms or competitive shielding, reducing immediate lethality and allowing for recovery analysis.

During the subsequent 24 h, *S. Enteritidis* continued to decline and became undetectable by 40 h (*P* < 0.05). In contrast, *B. subtilis* gradually recovered after 32 h and stabilized above 8.0 log CFU/mL, highlighting distinct post-treatment trajectories between the two strains. These results demonstrate that *B. subtilis* exhibits not only superior initial tolerance to SAEW but also a stronger capacity for post-stress recovery in a mixed microbial environment. The sustained decline of *S. Enteritidis*, even in the presence of a cohabiting species, suggests a lack of effective repair or compensatory mechanisms. This divergence highlights that while SAEW initially imposes broad-spectrum inactivation, long-term microbial outcomes are shaped by intrinsic physiological resilience.

Previous studies have indicated that SAEW exerts its bactericidal effect primarily through oxidative stress mediated by HClO (7, 8). However, different bacterial species respond variably depending on their cellular structures and stress adaptation pathways. For example, Lee et al. (31) reported that certain lactic acid bacteria could recover viability after oxidative disinfection by activating membrane repair systems and stress-responsive genes, whereas susceptible pathogens showed irreversible damage. In our study, the robust recovery of *B. subtilis* may reflect activation of similar protective mechanisms.

Notably, *B. subtilis* is known to employ PerR-regulated antioxidant defenses under oxidative pressure and may utilize the σᴮ-dependent general stress response under broader stress conditions (21–23). This regulatory system modulates the expression of antioxidant enzymes and molecular chaperones to mitigate ROS accumulation and maintain cellular homeostasis (21). Additionally, some studies suggest that *B. subtilis* can transiently enter a pre-spore metabolic state without full sporulation, downregulating energy-intensive pathways to survive extreme conditions (32). Such adaptive strategies may account for the observed delayed but sustained recovery phase.

In contrast, *S. Enteritidis*, a Gram-negative pathogen, is particularly vulnerable to oxidative agents due to its thinner cell envelope and less efficient ROS detoxification systems (24, 33). SAEW-induced damage, including lipid peroxidation, ATP depletion, and protein leakage, has been shown to cause irreversible cellular failure in this species (9, 24). Its continued decline in our study, despite an initial transient rebound, reinforces its inability to cope with sustained oxidative insult.

Although SAEW is widely regarded as a broad-spectrum disinfectant, Zhao et al. (28) have noted its selective antimicrobial behavior under certain conditions. They reported that SAEW vaginal irrigation effectively suppressed pathogenic anaerobes while sparing beneficial lactobacilli. Our findings support this potential selectivity: although *B. subtilis* viability dropped by over 2 log units immediately post-treatment, its ability to recover and reestablish a stable population suggests functional resilience. This highlights the dual challenge of using SAEW in practical applications: achieving pathogen elimination while avoiding excessive suppression of beneficial strains.

These results confirm the reproducibility of interspecies tolerance differences under dynamic conditions and establish a foundation for mechanistic investigation. Therefore, we next sought to explore the cellular and physiological mechanisms that enable *B. subtilis* to survive and recover from SAEW-induced oxidative stress, while *S. Enteritidis* fails to do so.

### Mechanisms underlying differences in tolerance of *B. subtilis* and *S. enterica* to SAEW stress

#### Disruption of cell membrane integrity and leakage of intracellular contents under SAEW stress

The bacterial cell membrane serves as a fundamental structural barrier, essential for maintaining intracellular homeostasis. Damage to the membrane can lead to the leakage of critical intracellular components, such as nucleic acids and proteins, disrupting cellular metabolism and ultimately resulting in cell death (34). To evaluate the impact of SAEW on membrane integrity, extracellular levels of nucleic acids and proteins were measured in both *B. subtilis* and *S. Enteritidis* before and after treatment.

**Fig 5:**
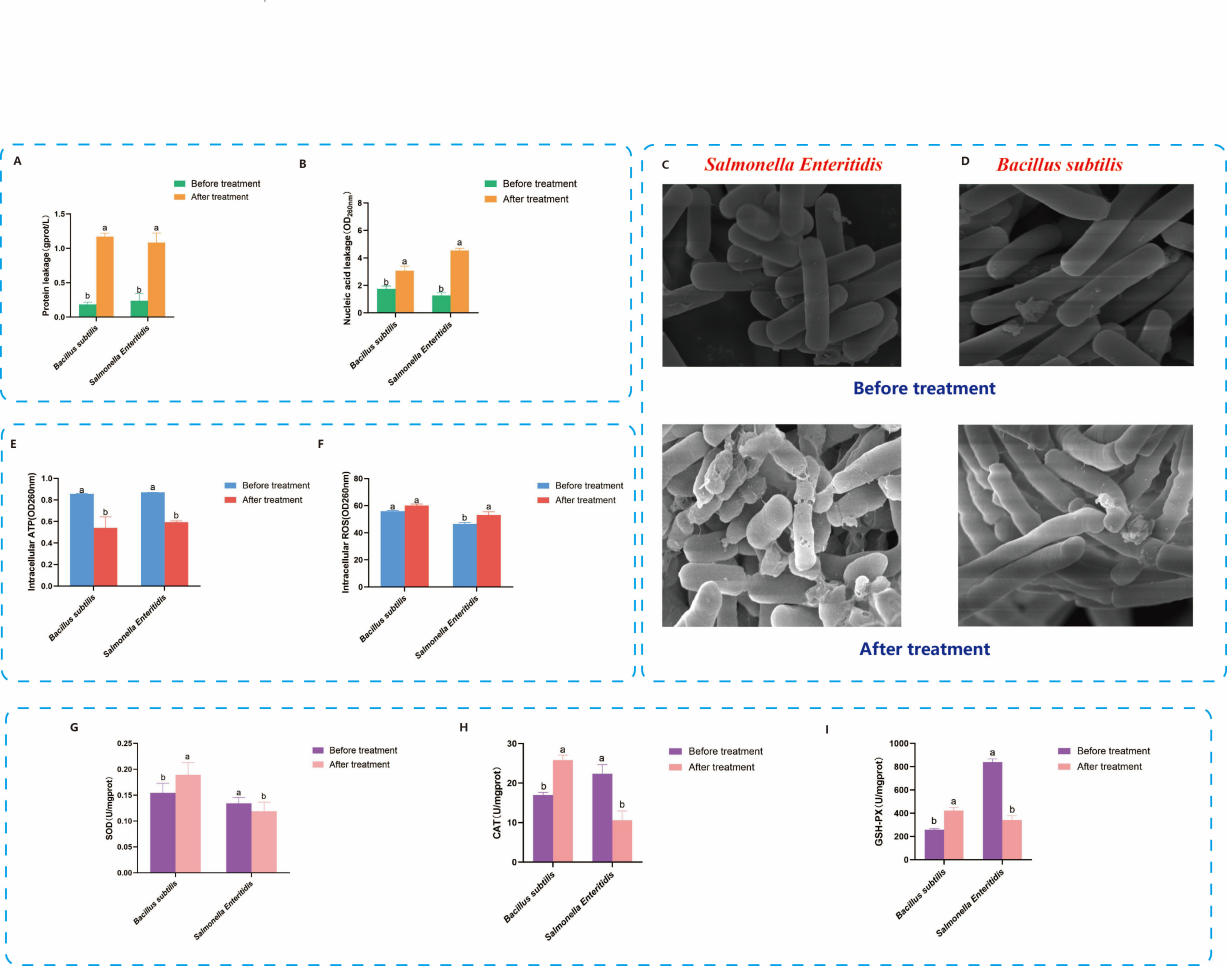
Comparative analysis of tolerance mechanisms of *Bacillus subtilis* and *Salmonella Enteritidis* under slightly acidic electrolyzed water stress. (**A**) Intracellular nucleic acid leakage before and after SAEW treatment. (**B**) Intracellular protein leakage before and after SAEW treatment. (**C**) Scanning electron microscopy (SEM) images showing morphological changes in *Salmonella Enteritidis* before and after SAEW treatment. (**D**) SEM images in *Bacillus subtilis* before and after SAEW treatment. (**E**) Intracellular ATP content before and after SAEW treatment. (**F**) Reactive oxygen species (ROS) levels before and after SAEW treatment. (**G–I**) Antioxidant enzyme activities (SOD, CAT, and GSH-Px) levels before and after SAEW treatment. Different lowercase letters indicate significant differences between groups (*P* < 0.05).

As shown in Fig. 5A and B, the results show that SAEW treatment significantly increased the extracellular concentrations of nucleic acids and proteins in both bacterial species, indicating membrane damage (*P* < 0.05). Notably, the extent of leakage was substantially greater in *S. Enteritidis* than in *B. subtilis*, suggesting a higher susceptibility of its membrane structure to SAEW. This differential response likely stems from intrinsic structural differences. *S. Enteritidis*, a Gram-negative bacterium, possesses an outer membrane rich in unsaturated fatty acids and lipopolysaccharides, making it more vulnerable to oxidative attack by HClO, the main active component in SAEW (24, 29). In contrast, *B. subtilis*, a Gram-positive bacterium, has a thicker peptidoglycan layer and a membrane enriched with saturated fatty acids, offering enhanced structural protection and contributing to its superior membrane stability (32).

To further validate these findings, scanning electron microscopy (SEM) was employed to examine the morphological changes in bacterial cells following SAEW exposure. As shown in Fig. 5C and D, the SEM images reveal that *S. Enteritidis* cells exhibited extensive surface deformation, including shrinkage, roughness, and even lysis. In contrast, most *B. subtilis* cells retained their structural integrity, with only minor dimpling or surface irregularities observed in a small subset. These observations confirm that while SAEW induces membrane damage in both species, *B. subtilis* exhibits greater morphological resilience, likely due to its more robust cell wall structure.

Previous studies support these conclusions. Fukuzaki (35) reported that HClO causes drastic alterations in cell surface morphology, while Li et al. (36) demonstrated that HClO disrupts the carbon chains of membrane phospholipids, reducing membrane fluidity and integrity. Although Ding et al. (37) and Zhang et al. (9) observed that *S. aureus* exhibited membrane wrinkling without evident leakage after SAEW exposure, the present study indicates that *B. subtilis* does experience partial membrane damage. Taken together, the results suggest that cell membrane integrity is a key determinant of the differential tolerance to SAEW stress between *B. subtilis* and *S. Enteritidis*.

#### Changes in intracellular ATP levels under SAEW stress

As shown in Fig. 5E, intracellular ATP levels significantly decrease in both *B. subtilis* and *S. Enteritidis* following SAEW treatment, with *B. subtilis* decreasing from 0.86 to 0.54 units and *S. Enteritidis* decreasing from 0.87 to 0.59 units (*P* < 0.05), consistent with previous findings. As a fundamental energy currency in living cells, ATP is essential for maintaining cellular homeostasis, and its depletion has been closely associated with cell damage or death (38). The observed reduction may result from increased membrane permeability caused by HClO and ClO⁻ in SAEW, which facilitates the leakage of intracellular ATP. Additionally, Zhang et al. (9) reported that HClO can penetrate bacterial cells and oxidize components of the electron transport chain (ETC), thereby disrupting proton gradient formation and ATP synthesis. Disruption of ATP production not only undermines energy metabolism but may also induce metabolic imbalance, contributing to the accumulation of intracellular ROS (38). Given these interconnected effects, the ROS levels of both species were further examined to clarify the potential link between SAEW-induced energy disruption and oxidative stress responses.

#### Accumulation of ROS under SAEW stress

Disruption of ATP metabolism often leads to redox imbalance and contributes to the intracellular accumulation of ROS (38). To further investigate whether SAEW treatment induced oxidative stress, ROS levels in both *B. subtilis* and *S. Enteritidis* were measured before and after exposure.

As shown in Fig. 5F, *S. Enteritidis* exhibits a significant increase in intracellular ROS following SAEW treatment, rising from 46.59 to 53.2 units (*P* < 0.05). This observation aligns with prior findings by Yuan et al. (24), who demonstrated a similar ROS elevation in *Salmonella* upon SAEW exposure. The increase in ROS may result from both ATP depletion and direct oxidative damage caused by HClO, the primary active species in SAEW (36). HClO can oxidize components of the bacterial ETC, such as membrane-bound dehydrogenases, thereby inhibiting proton transport and accelerating ROS production (29, 36). Additionally, SAEW-induced membrane disruption can alter metal ion homeostasis and further promote oxidative imbalance.

In contrast, *B. subtilis* did not exhibit a significant increase in intracellular ROS after SAEW treatment (*P* > 0.05). This finding differs from previous observations in other Gram-positive bacteria, such as *S. aureus*, where SAEW-induced inactivation is accompanied by increased ROS levels (37). The absence of ROS accumulation in *B. subtilis*, despite a noticeable drop in ATP, suggests the presence of an effective antioxidant defense system that mitigates ROS accumulation and enhances stress tolerance. This is consistent with the greater survival and recovery capacity observed in *B. subtilis* compared to *S. Enteritidis* in earlier sections.

As ROS plays a central role in determining cell fate under oxidative stress, and given the species-specific differences in ROS accumulation patterns (23, 25), the next section focuses on characterizing the activities of key antioxidant enzymes, particularly SOD and CAT, to clarify their roles in mediating resistance to SAEW.

#### Differential responses of antioxidant systems under SAEW stress

Under oxidative stress, enzymatic antioxidant systems serve as the first line of defense for bacteria to maintain redox homeostasis and mitigate ROS-induced damage (22, 23). As shown in Fig. 5G through I, in this study, SAEW exposure markedly disrupts the antioxidant defense in *S. Enteritidis*, as evidenced by significant reductions in intracellular SOD, CAT, and GSH-Px activities. Specifically, SOD activity decreased from 0.134 ± 0.01 to 0.119 ± 0.018 U/mg protein (*P* < 0.05), CAT from 22.36 ± 2.3 to 10.61 ± 2.37 U/mg protein (*P* < 0.001), and GSH-Px from 839.6 ± 27.7 to 341.7 ± 37.86 U/mg protein (*P* < 0.05). These results indicate that SAEW treatment severely impaired the ability of *S. Enteritidis* to scavenge intracellular ROS, leading to redox imbalance and cumulative cellular damage.

In contrast, *B. subtilis* exhibited a distinctly upregulated antioxidant response. Following SAEW exposure, SOD activity significantly increased from 0.15 ± 0.02 to 0.19 ± 0.02 U/mg protein (*P* < 0.05), CAT activity increased from 16.98 ± 0.66 to 25.81 ± 1.28 U/mg protein (*P* < 0.05), and GSH-Px from 258.82 ± 8.85 to 424 ± 24.59 U/mg protein (*P* < 0.05). These enzyme activities not only surpassed untreated controls but, in some cases, exceeded those of *S. Enteritidis* even prior to SAEW treatment, reflecting a robust and inducible antioxidant system capable of counteracting oxidative stress.

This difference may be attributed to distinct redox regulatory mechanisms. In *B. subtilis*, previous studies have suggested that the peroxide-responsive repressor PerR plays a central role in oxidative stress adaptation (22, 23). Upon exposure to H₂O₂, oxidation of PerR-bound Fe²^+^ reportedly derepresses genes such as *sodA*, *katA*, and *ahpC*, thereby promoting the production of ROS-scavenging enzymes (22, 23). Although the present study did not directly assess PerR activity, the coordinated upregulation of antioxidant enzymes observed in *B. subtilis* under SAEW exposure supports the possibility that such transcriptional regulation contributes to its enhanced stress resilience. Further investigation is warranted to determine whether PerR or related regulons (e.g., Spx or SigB pathways) are activated in response to SAEW-induced oxidative stress. Moreover, the upregulation of antioxidant enzymes in *B. subtilis* occurred despite a concurrent drop in ATP levels, suggesting that *B. subtilis* can reallocate metabolic energy toward defense responses even under energy-limiting conditions, a potential strategy for persistence in hostile environments (22, 23).

**Fig 6:**
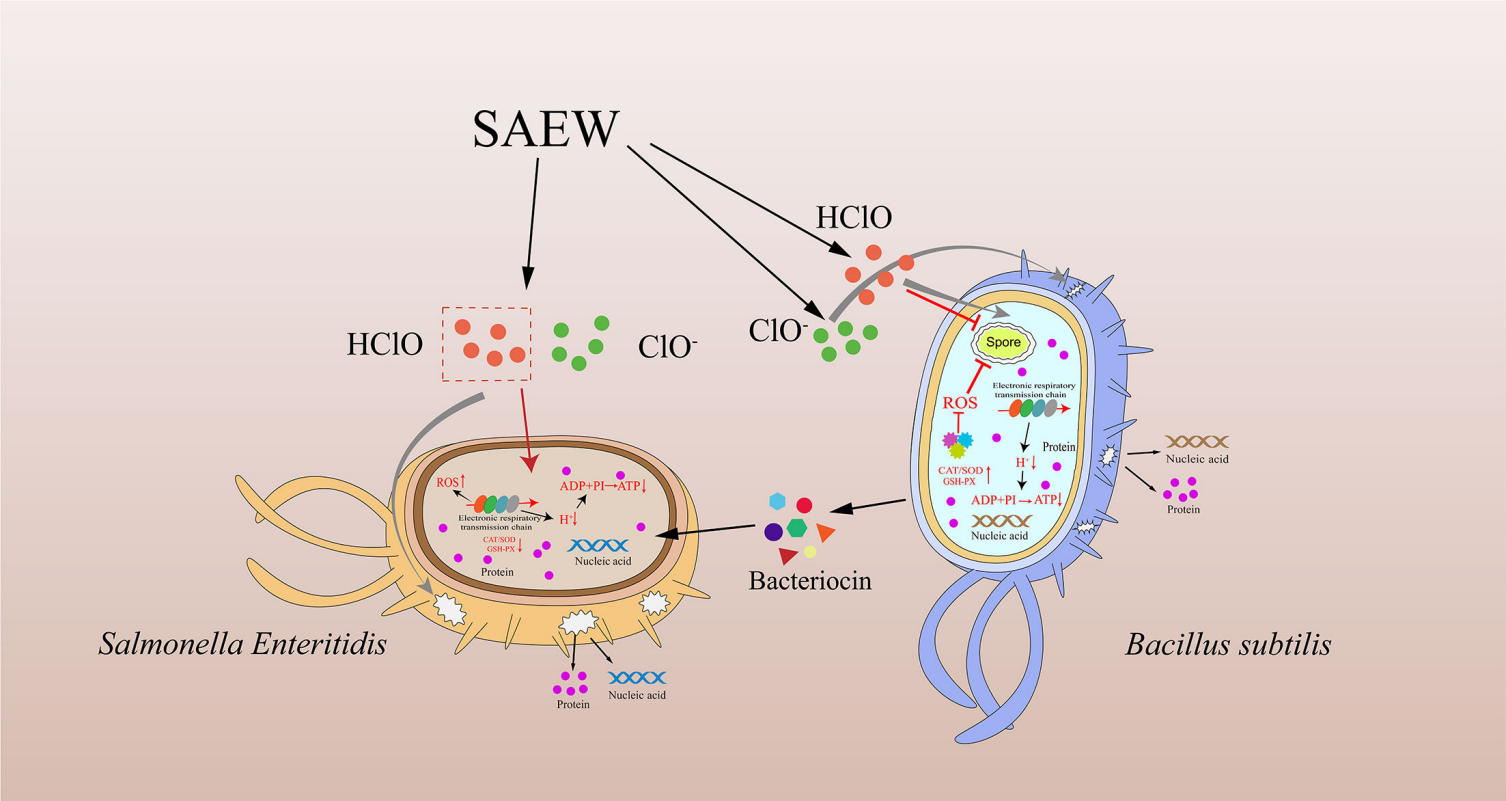
Proposed schematic of differential tolerance mechanisms of *Bacillus subtilis* and *Salmonella Enteritidis* under slightly acidic electrolyzed water (SAEW) stress. *B. subtilis* shows enhanced oxidative stress response via elevated antioxidant enzyme activity (SOD, CAT, GSH-Px) and possible sporulation-based protection, while *S. Enteritidis* exhibits membrane damage, intracellular content leakage, ROS accumulation, and disruption of ATP synthesis. SAEW-generated HClO and ClO⁻ are key contributors to the observed cellular damage. This schematic integrates physiological, biochemical, and structural responses under SAEW-induced oxidative stress.

Taken together, these findings confirm that differential antioxidant system activation plays a decisive role in determining bacterial survival under SAEW stress. The rapid suppression of enzyme activity in *S. Enteritidis* contrasts with the inducible fortification observed in *B. subtilis* and may explain the distinct cell fate trajectories illustrated in Fig. 6.

#### Integrated interpretation of SAEW tolerance mechanisms and limitations

The results of this study demonstrate that *B. subtilis* and *S. Enteritidis* exhibit markedly distinct survival trajectories under SAEW-induced stress, as summarized in Fig. 6. SAEW triggered a cascade of physiological disturbances—including membrane damage, ATP depletion, ROS accumulation, and disruption of antioxidant defenses—while the coordination of bacterial responses to these insults ultimately determined their survival or death.

In *S. Enteritidis*, SAEW exposure caused extensive membrane disruption, as evidenced by substantial leakage of intracellular proteins and nucleic acids, with SEM revealing collapsed cell structures. Concomitantly, intracellular ATP levels declined sharply, ROS levels rose significantly, and antioxidant enzyme activities (SOD, CAT, GSH-Px) were markedly suppressed. These findings suggest that *S. Enteritidis* lacks an effective defense mechanism against high-intensity oxidative stress.

In contrast, *B. subtilis* exhibited a more resilient response. Despite experiencing similar membrane damage and ATP reduction, its intracellular ROS levels remained stable, and antioxidant defenses were significantly upregulated. This robust response may be attributed to specialized regulatory systems, such as PerR, Spx, and SigB, which have been implicated in redox homeostasis in *B. subtilis* (23, 39). However, this resilience may primarily be mediated by the PerR-regulated antioxidant system, which rapidly upregulates ROS-scavenging enzymes (SOD, CAT, GSH-Px) to mitigate oxidative damage (22, 23). While ancillary regulators such as Spx (for thiol homeostasis) and SigB (for general stress proteins) may provide supplementary protection, PerR constitutes the frontline defense against SAEW-derived peroxides. Additionally, structural features, such as a thicker peptidoglycan cell wall and the capacity for sporulation, may enhance *B. subtilis* physical stability and stress endurance (39, 40).

This study also presents several limitations. Although PerR and associated regulators are hypothesized to underlie the differential antioxidant responses in *B. subtilis*, their transcriptional or translational dynamics were not directly measured. Verification through omics-based approaches or gene knock-out experiments would strengthen mechanistic understanding. Moreover, while the co-culture model captures competitive dynamics between the two strains, it does not fully reflect the complexity of multispecies microbial ecosystems. Future studies should incorporate more ecologically relevant conditions, such as biofilm formation and microbial succession, to better evaluate microbial behavior under disinfection stress.

### Conclusion

This study systematically compared the survival differences between *B. subtilis* and *S. Enteritidis* under SAEW stress, as well as the underlying mechanisms. Through a series of experiments involving monoculture, co-culture, and post-treatment dynamic responses, *B. subtilis* consistently exhibited greater resistance than *S. Enteritidis*. Notably, *B. subtilis* remained viable even at 70 mg/L ACC, from 7.82 to 3.99 log CFU/mL (*P* < 0.05), whereas *S. Enteritidis* was completely inactivated at 40 mg/L. In the co-culture system, following 50 mg/L SAEW treatment, *B. subtilis* maintained significantly higher viable counts than *S. Enteritidis* (*P* < 0.05), indicating a competitive advantage under shared ecological conditions. Furthermore, *B. subtilis* showed a stronger capacity for post-SAEW stress recovery in a mixed microbial environment than *S. Enteritidis*. SAEW exposure led to membrane disruption and leakage of intracellular proteins and nucleic acids in both species. Subsequent measurements revealed that while ATP levels declined in both strains, *S. Enteritidis* exhibited a pronounced accumulation of ROS and a significant suppression of antioxidant enzymes (*P* < 0.05). In contrast, *B. subtilis* showed marked upregulation of SOD, CAT, and GSH-Px (*P* < 0.05) and no significant ROS accumulation (*P* > 0.05), demonstrating a stronger oxidative stress response and cellular stability under SAEW stress. Collectively, these findings indicate that SAEW exerts selective pressure that efficiently eliminates pathogenic bacteria while partially sparing stress-tolerant probiotics, such as *B. subtilis*, providing a theoretical basis for its targeted application in food processing and livestock production. Future studies integrating transcriptomics or gene-editing approaches are warranted to further elucidate the molecular mechanisms underlying the differential tolerance between these species.
